# Evaporative deposition of polystyrene microparticles on PDMS surface

**DOI:** 10.1038/s41598-017-14593-5

**Published:** 2017-10-26

**Authors:** Ying-Song Yu, Ming-Chao Wang, Xianfu Huang

**Affiliations:** 1 Department of Mechanics, School of Civil Engineering, Architecture and Environment, Hubei University of Technology, Wuhan, 430068 China; 2 State Key Laboratory of Nonlinear Mechanics, Institute of Mechanics, Chinese Academy of Sciences, Beijing, 100190 China; 3 School of Engineering Science, University of Chinese Academy of Sciences, Beijing, 100049 China

## Abstract

Evaporation of water and ethanol/water droplets containing large polystyrene (PS) microparticles on polydimethylsiloxane (PDMS) surface was experimentally investigated. It is found that no matter with or without small addition of ethanol, a compact monolayer deposition is formed for lower microparticle concentration while mountain-like deposition for higher concentration. Since the more volatile compound (ethanol) evaporates more quickly than the less volatile compound (water), evaporation of ethanol/water mixture droplet exhibits different characteristics from pure water. When the concentration of microparticle is low, the contact radius of ethanol/water mixture droplet decreases throughout the whole process, while the contact angle increases at first to a maximum, then keeps almost constant, and finally decreases sharply. However, the evaporation of ethanol/water mixture droplet with higher concentration of microparticle behaviors more complex. The settling time of microparticles was estimated and its theoretical value agrees well with the experimental one. Moreover, a mechanism of self-pinning of microparticles was used to elucidate the deposition behavior of microparticles, indicating that as the contact line is depinning, the liquid film covering the outmost microparticle becomes thicker and thicker, and the microparticles have to move spontaneously with the depinning contact line under the action of capillary force.

## Introduction

When a sessile suspension droplet containing micro/nano-particles evaporates on a solid substrate, these particles will be left on the substrate after evaporation. Using this method, we can obtain desirable deposition pattern for applications in fields such as electronics, optoelectronics, sensors, nanotechnology, and biotechnology, etc.^1^ However, when the substrate is hydrophilic and the contact line is pinned, there will be a singular evaporation flux near the contact line, resulting in a strong outward capillary compensation flow (it should be noted that there is no singularity of the evaporation flux near the contact line for hydrophobic case and thus the outward capillary flow is very weak), which will carry the particles (whose diameter is usually of the order of several micrometers or below) towards the edge. Then a ring-like structure is formed at the perimeter, which is known as the coffee-ring effect^2^. Besides of the contact line pinning, suppression of Marangoni flow is another necessary condition for the formation of the coffee ring^3^. Shen *et al*.^4^ pointed out that there will be no coffee ring effect when the liquid evaporates much faster than the movement of particle, and found that a coffee ring structure will be still formed until the droplet size decreases to a critical value. Marín *et al*.^5^ showed that the singularity of the flow velocity at the end of an evaporating droplet will bring about a sharp transition from ordered crystals to disordered packings in the coffee stain. The coffee-ring phenomenon^6–8^ makes the deposition pattern nonuniform and thus hinders the application of droplet evaporation. Moreover, using droplet evaporation, except for coffee ring structure, researchers obtained different deposition patterns such as hexagon^9,10^, stripe^10^, hemispherical particle assemblies with ordered nanoporous structures^11^, highly ordered monolayer^12,13^, concentric ring^14^, coffee eye^15^, inner deposit^16^ and nearly uniform deposition^17,18^.

To better understand the coffee-ring phenomenon and apply evaporation-induced deposition, researchers have set up theoretical models^19–24^ for self-pinning of microparticles at the contact line. Using the scaling analysis, Jung *et al*.^19^ estimated the role of various forces such as drag, electrostatic, van der Waals, and capillary on the particle motion and found that i) the motion of a single particle suspended in liquid is mainly affected by drag force in the pinned contact line stage, and ii) capillary force controls its motion in the depinning contact line stage. Wong *et al*.^20^ elucidated the physics of particle separation during coffee-ring formation based on a particle-size selection mechanism near the contact line of an evaporating droplet. Later, on the basis of Jung *et al*.^19^ and Wong *et al*.^20^, Chhasatia and Sun^21^ proposed a self-pinning mechanism, however, the velocity of water in evaporating drop 0.2 m/s was suspectable. In 2013, Weon and Je^22^ compared the spreading and drying behaviors of pure and colloidal droplets and set up a mechanism for the self-pinning of particles at the contact line. Hurth *et al*.^23^ conducted evaporation of sessile droplet containing streptavidin- or biotin-coated fluorescent polystyrene (PS) particles and found that both the biological binding force and the capillary force play significant roles in particle deposition and that the viscous drag, van der Waals forces, and solid-solid friction forces are negligible. Recently, using particle tracking velocimetry technique, Yu *et al*.^24^ studied the motion of fluorescent PS microparticles at the depinning contact line and built up a self-pinning mechanism. Besides, Li *et al*.^25^ predicted three different motions of a single nanoparticle at the contact line using molecular dynamics simulation.

Besides single-component droplet, mixture droplet has also been widely used in many fields. Because different component of a mixture droplet usually has different evaporation rate and there is a diffusion of one component into another, evaporation of a mixture droplet is more complex. For example, evaporation of water/1-propanol mixture droplets at room temperature on polymethyl methacrylate (PMMA) exhibits two types of behavior depending on the molar fraction of the mixture^26^. One is a long-time contact line pinning (scaled around two thirds of the evaporation time) for the molar fracture greater than the azeotropic point (0.39 mol fraction of 1-propanol), and the other is an instable behaviour when its molar fraction is less than the point. Rusdi *et al*.^27^ found that the evaporation rate for water-ethylene glycol liquid mixture increases with increasing temperature, and decreases with increasing mole fraction of ethylene glycol. Evaporation of ethanol/water mixture droplets has been extensively studied on smooth planer surface^28–31^ and chemical micro-patterned surface^32^. And it is found that the contact line recedes throughout the evaporation and the contact angle increases at first to a maximum and decreases thereafter. Christy *et al*.^31^ found that there are three evaporating stages of ethanol/water droplet on clean glass surface, *viz*., a multiple-vortices-dominated stage, a transition stage due to a remarkable spike in outward flow, and a stage because of outward flow which is the same to that of pure water. Therefore, evaporation of mixture droplet might be used to eliminate the coffee ring effect.

**Figure 1:**
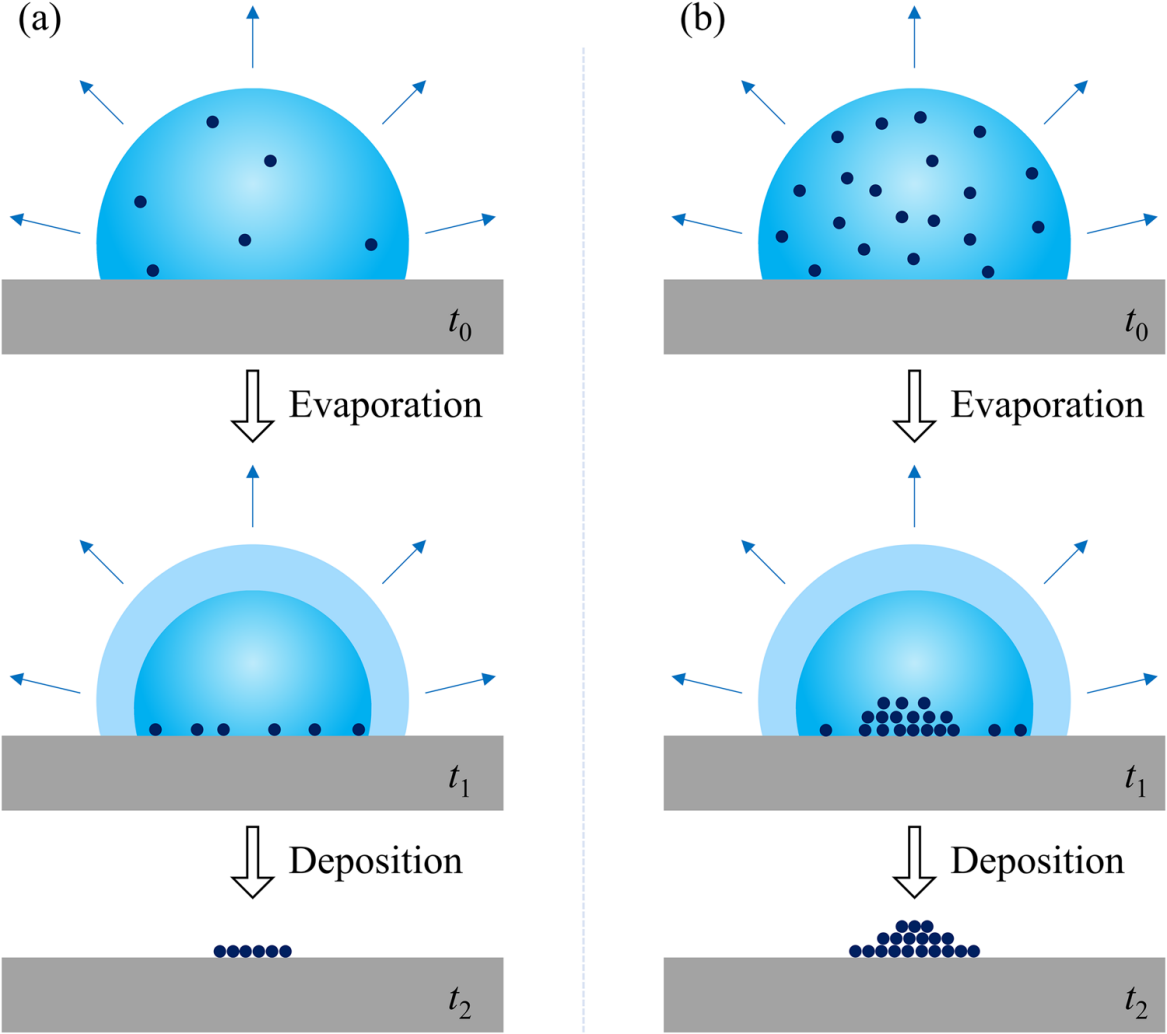
Schematics of evaporative deposition on PDMS surface showing the settling of microparticles and the evaporative deposition. After evaporation, a monolayer (**a**) is formed for lower microparticle concentration while mountain-like deposition (**b**) for higher concentration.

Due to good biocompatibility, nontoxicity, optical transparency and easy fabrication, polydimethylsiloxane (PDMS) has been widely used in micro- and nano-systems. Recently, sessile droplet on PDMS surface has been intensively studied and it is found that substrate deformation induced by sessile droplet significantly influences the wetting and evaporation characteristics as well as the deposition pattern after evaporation^33–38^. Weon and He^39^ reported that the coffee-ring effect can be suppressed for the case of sessile evaporating droplet containing large microparticle under the action of capillary force. In this paper, evaporation of sessile water and ethanol/water droplets containing PS microparticles with diameter of 20.33 μm was experimentally investigated. When the concentration of microparticle is low, the contact radius of ethanol/water mixture droplet decreases throughout the whole process, while the contact angle increases at first to a maximum, then keeps almost constant, and finally decreases sharply. However, the evaporation of ethanol/water mixture droplet with higher concentration of microparticle behaviors more complex. Settling time of microparticles inside evaporating droplet was estimated and compared with the experimental data. Finally, deposition patterns were analyzed using a laser scanning confocal microscope. It was found that i) at low particle concentration, a compact monolayer deposition was formed while compact multilayer structures was formed for high concentration, ii) addition of ethanol has no significant influence on deposition pattern, as illustrated in Fig. 1. Moreover, the formation of deposition patterns was elucidated using a self-pinning mechanism of microparticles confined at the contact line^24^.

## Results

### Evaporation of sessile droplet

**Figure 2:**
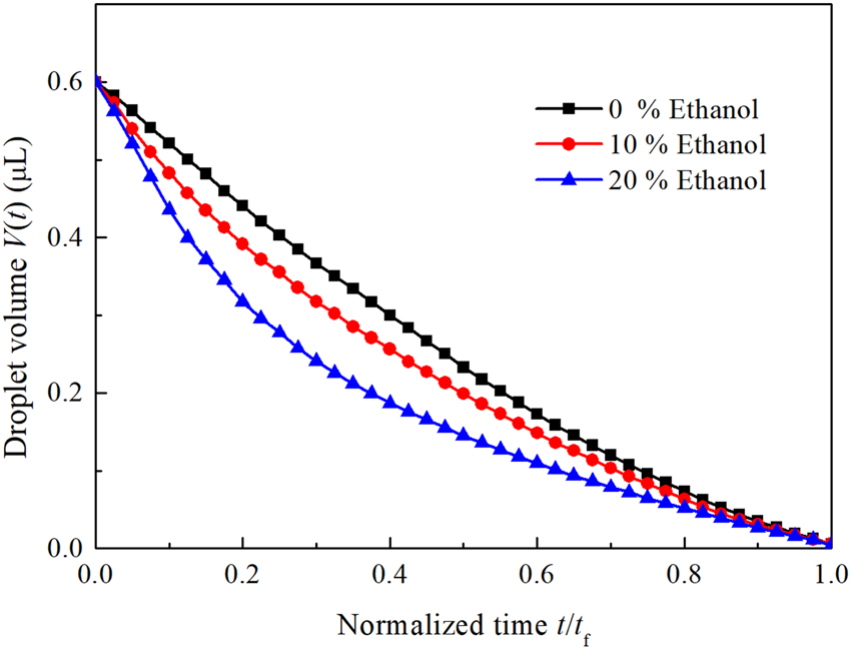
Droplet volume versus time for water and ethanol/water droplets containing 0.02 wt.% PS microparticles.

Because the characteristic length of 0.6 μL sessile droplet is obviously less than its capillary length, the influence of gravity on its shape is negligible and the droplet has a spherical cap. Thus the volume can be written as

1 $$V=\frac{\pi {a}^{3}{(1+\cos \theta )}^{2}(2-\cos \,\theta )}{3\,{\sin }^{3}\theta },$$

where $V$, $a$ and $\theta$ are the volume, contact radius and contact angle, respectively. Substituting the measured initial contact angles into Eq. (1), the initial contact radii were obtained. Both the contact radius and the contact angle have been averaged. Figure 2 shows the evolution of volume of pure water droplet and ethanol/water mixture droplet containing 0.02 wt.% PS microparticles versus normalized time $t/{t}_{{\rm{f}}}$ (${t}_{{\rm{f}}}$ is the total evaporation time) (the curves for droplets containing 1.28 wt.% PS microparticles are not given because each of them is similar to that for 0.02 wt.% case). When the concentration of ethanol is low (10%), the slope of the droplet volume differs slightly from that of pure water. As the concentration of the volatile liquid increases to 20%, the curve deviates from that of pure water. As compared with the slope for pure water, that for mixture droplet becomes larger at first and less later when ethanol is introduced into the droplet.

**Figure 3:**
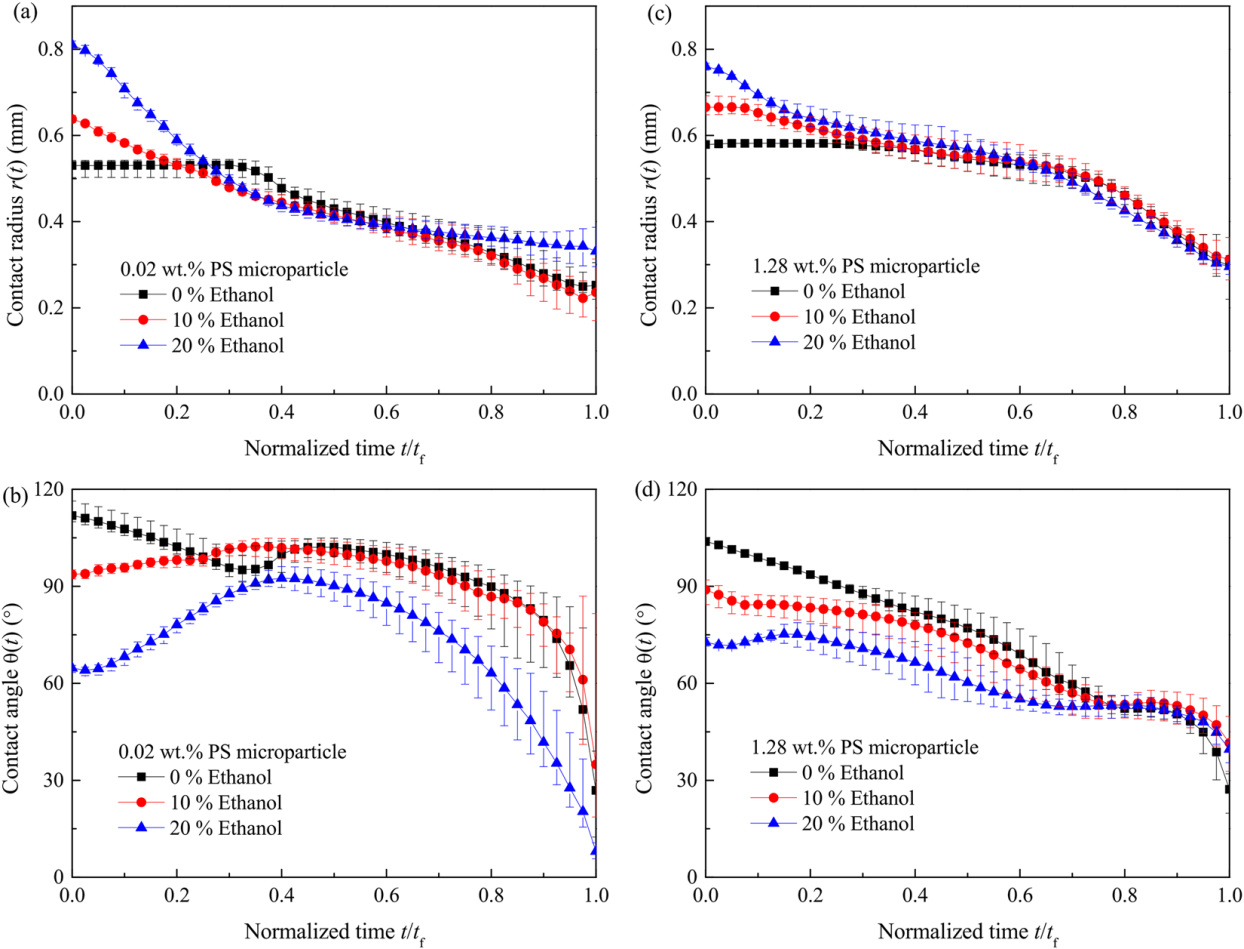
Evaporation curves of sessile water and ethanol/water droplets containing 0.02 wt.% PS microparticles (**a** contact radius, **b** contact angle) and 1.28 wt.% PS microparticles (**c** contact radius, **d** contact angle).

Figure 3 shows the evolution of contact radius and contact angle versus time for pure water droplet and ethanol/water mixture droplet containing 0.02 and 1.28 wt.% PS microparticles. For water droplet containing colloidal particles, due to contact angle hysteresis, all experiments proceeded with constant contact radius (CCR) mode, constant contact angle (CCA) mode and mixed mode. For ethanol/water mixture droplet, because the vapor pressure of ethanol is about 7.0 kPa at 23 °C, which is higher than that of water (~2.8 kPa at 23 °C)^29^, ethanol will evaporates faster and the surface tension of mixture is increased. Meanwhile, the diffusion coefficient of ethanol into water is about $1.22\times {10}^{-9}{{\rm{m}}}^{{\rm{2}}}/s$^40^, which is much smaller than that of ethanol into air ($\sim 1.2\times {10}^{-5}{{\rm{m}}}^{{\rm{2}}}/s$^41^). Thus the diffusion of inside the mixture droplet also controls the evaporation of ethanol from the droplet. As more and more ethanol evaporates from the liquid-vapor interface, the evaporation of ethanol/water mixture droplet behaviors as that of pure water droplet at the final stage. For mixture droplet containing 0.02 wt.% PS microparticles, the contact radius decreases throughout the whole process, while the contact angle increases at first to a maximum and decreases later. However, when the microparticle concentration of mixture suspension droplet increases to 1.28 wt.%, the contact angle exhibits different behavior. For 10% ethanol, at first there is short CCR stage and then both the contact radius and the contact angle decrease. After it, the contact angle keeps almost constant and finally the droplet evaporates completely with mixed mode. For 20% ethanol concentration, the contact radius decreases all over the whole process and the contact angle increases to a maximum at first. Then it decreases to about 53° and keeps almost constant for about 35% of the whole evaporation time. Finally, the evaporation completes with mixed mode.

**Figure 4:**
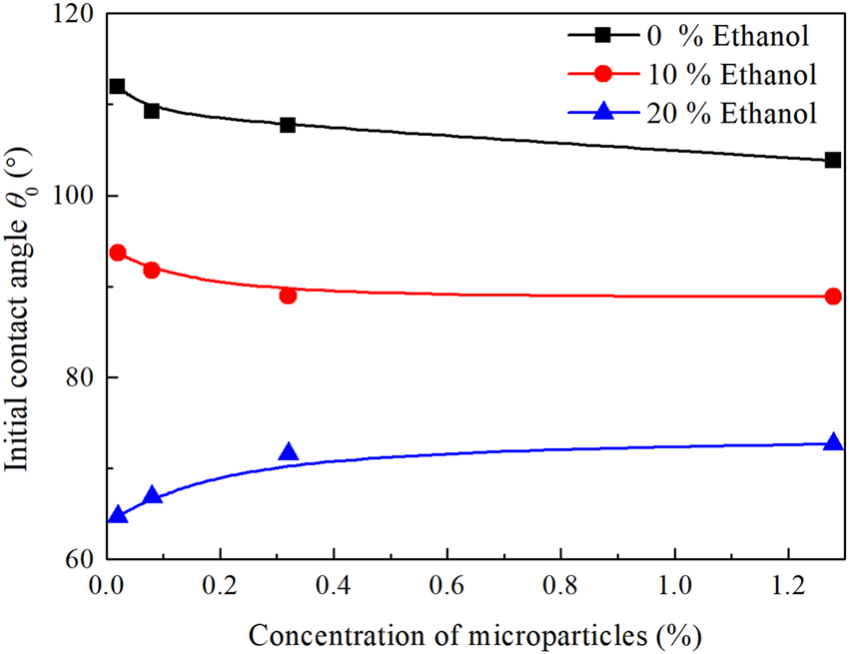
Initial contact angle for water mixtures droplet with different concentrations of PS microparticles on PDMS surface.

Figure 4 shows the initial contact angles of sessile droplets with different concentrations of ethanol and PS microparticles. On the one hand, at the same concentration of PS microparticles, the initial contact angle decreases with increasing ethanol concentration, which can be explained using Young’s equation. As ethanol was added into water, both the liquid-vapor and solid-liquid interfacial tensions decreased, thus the contact angle has to decrease. On the other hand, at the same ethanol concentration, the initial contact angle decreases with increasing concentration of PS microparticles for 0% ethanol and 10% ethanol while increases with increasing concentration of PS microparticles for 20% ethanol. The reason is not clear, and it might be related to the interactions between microparticles and water or ethanol, between water and ethanol, and between the mixture and the substrate, etc.

### Settling time of microparticles

The drag force acting on a microparticle by evaporating droplet can be calculated from the Stokes equation^42,43^ and given as

2 $${F}_{{\rm{d}}}=6\pi R\eta u$$

where *R* is the radius of the microparticle, *η* is the dynamic viscosity of water (~9 × 10^−4^ Pa·s), *u* is the velocity of the microparticle relative to the droplet in the vertical direction. Such a force is balanced by the net force between the gravity of the microparticle and its buoyancy as

3 $$F=\frac{4}{3}\pi ({\rho }_{P}-{\rho }_{L}){R}^{3}g$$

where ${\rho }_{P}$ is the particle density (1050 kg/m^3^), ${\rho }_{L}$ is the fluid density (1000 kg/m^3^), *g* is the gravitational acceleration (9.8 m/s^2^). Thus we can get the resulting settling velocity as

4 $${U}_{{\rm{S}}}=\frac{2}{9}\frac{({\rho }_{P}-{\rho }_{L}){R}^{2}g}{\eta }$$

**Figure 5:**
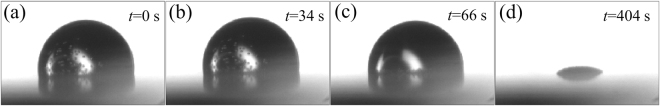
Images of evaporation of sessile water droplet containing 0.32 wt.% PS microparticle on PDMS surface. The visable dots insides the droplet in (**a,b**) represent the microparticles. (**a–c**) Shows the settling of microparticles inside the droplet and (**d**) shows the final deposition.

The calculated settling velocity for the microparticles inside water droplets is 13.8 μm/s. 0.60 μL pure water droplet with 0.02, 0.08, 0.32 and 1.28 wt.% PS microparticles on PDMS has, respectively, average initial droplet heights of 1.099, 1.066, 1.027 and 0.828 mm. Thus the times for all particles to settle are 79.6, 77.2, 74.4 and 60.03 s, respectively. From the videos (see supplementary information) we observed the motion of some microparticles inside pure water droplets containing PS microparticles. From (supplementary movies 1–4), we estimated the settling time for pure droplet containing 0.02, 0.08, 0.32 and 1.28 wt.% PS microparticles are, respectively, about 120, 90, 130 and 66 s. As an example, Fig. 5 shows the location of microparticles inside evaporating droplet at different time and it is found that there are more microparticles settling on the central zone of solid-liquid interface. For ethanol/water mixture droplets, because ethanol is more volatile, there will a more intense internal flow inside them than pure water droplet, which makes the settling of microparticles difficult. Under the action of such a flow, the microparticles inside the droplet moves so quickly that we cannot track their motion at the speed of 0.5 fps.

### Analysis of deposition patterns

**Figure 6:**
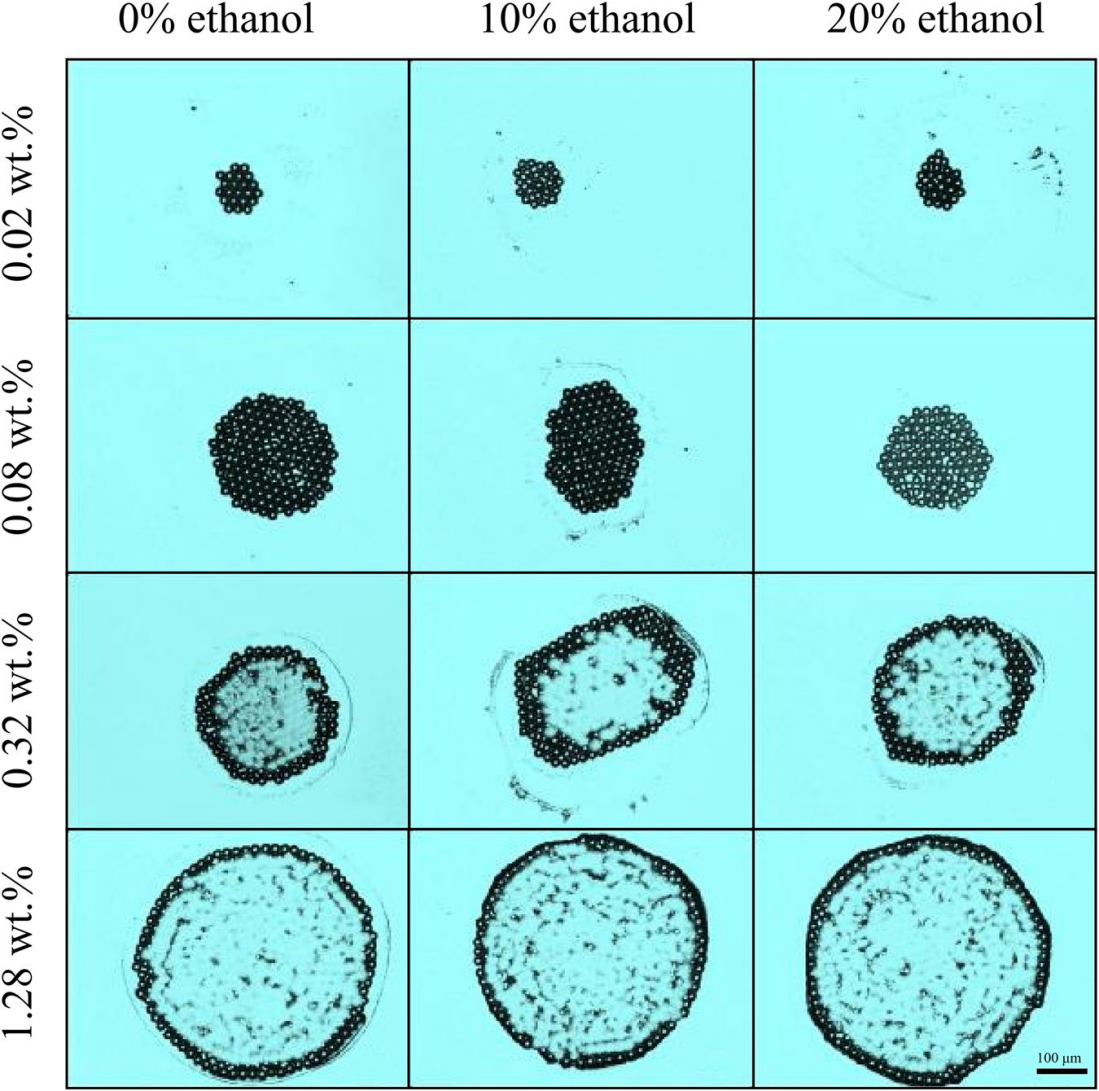
Deposition pattern of PS microparticles after evaporation.

**Figure 7:**
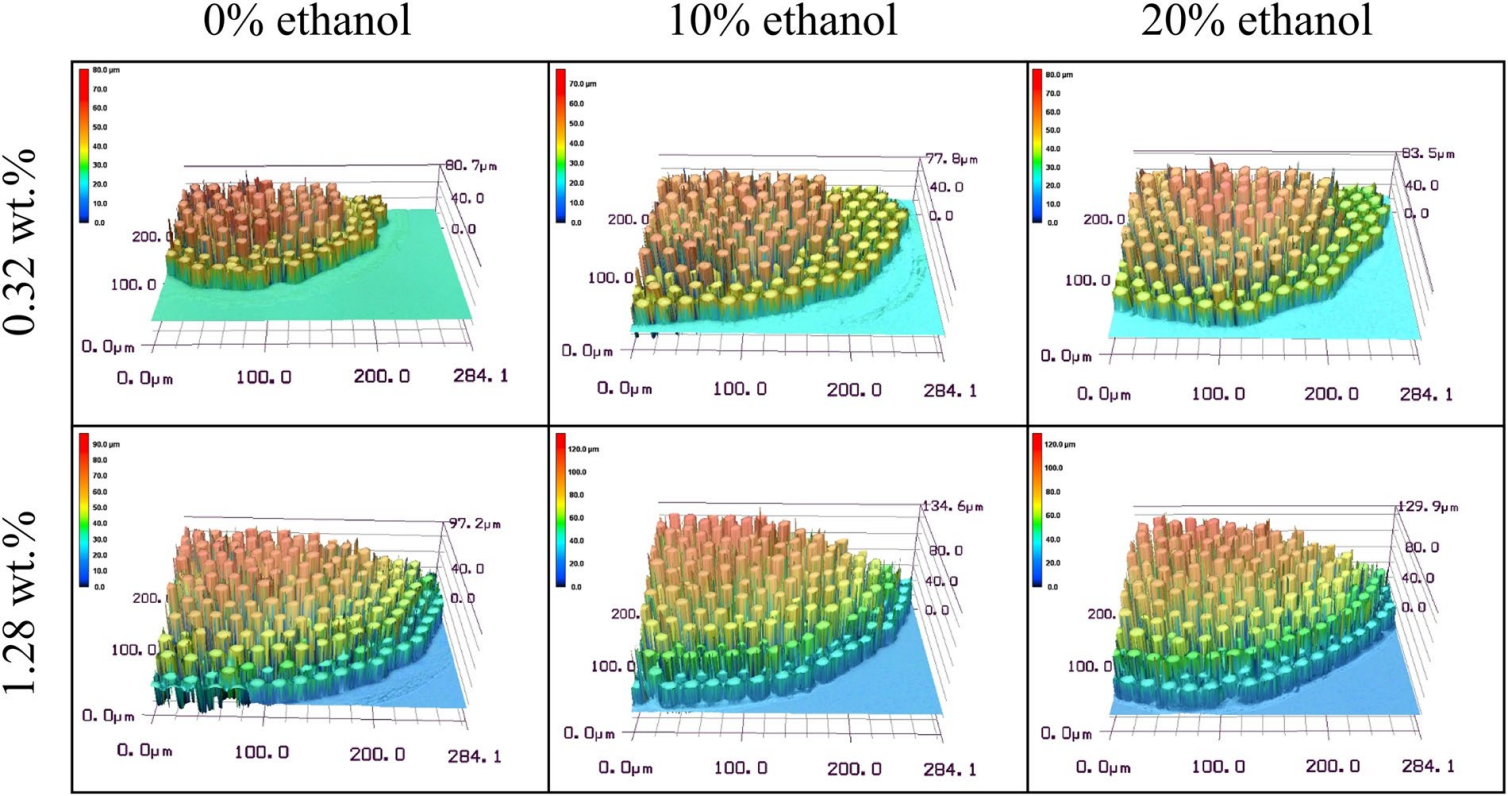
Deposition height of evaporation stains.

Figure 6 shows the deposition patterns of evaporating droplets with different microparticle concentration and ethanol concentration. To get a more detailed information on deposition patterns for higher microparticle concentration, we choosed about 1/4 of the deposition patterns and measured their topographical features (as shown in Fig. 7). It was found that 1) when the concentration of PS microparticles is low, there will be a compact monolayer pattern while multi-layered or mountain-like deposition is formed for higher microparticle concentration, 2) the addition of ethanol into the liquid droplet has an influence on the shape of the deposition pattern.

Why is there a compact monolayer deposition for lower microparticle concentration and a compact multi-layer structure for higher one? As Fig. 3 shows, no matter pure water droplet or ethanol/water mixture droplet, the depinning contact line stage dominates the droplet evaporation. When all the microparticles settle on PDMS surface, the outmost microparticles will experience van der Waals force, electrostatic force, drag force and capillary force when the receding three-phase contact line (TPCL) approaches to them.

**Figure 8:**
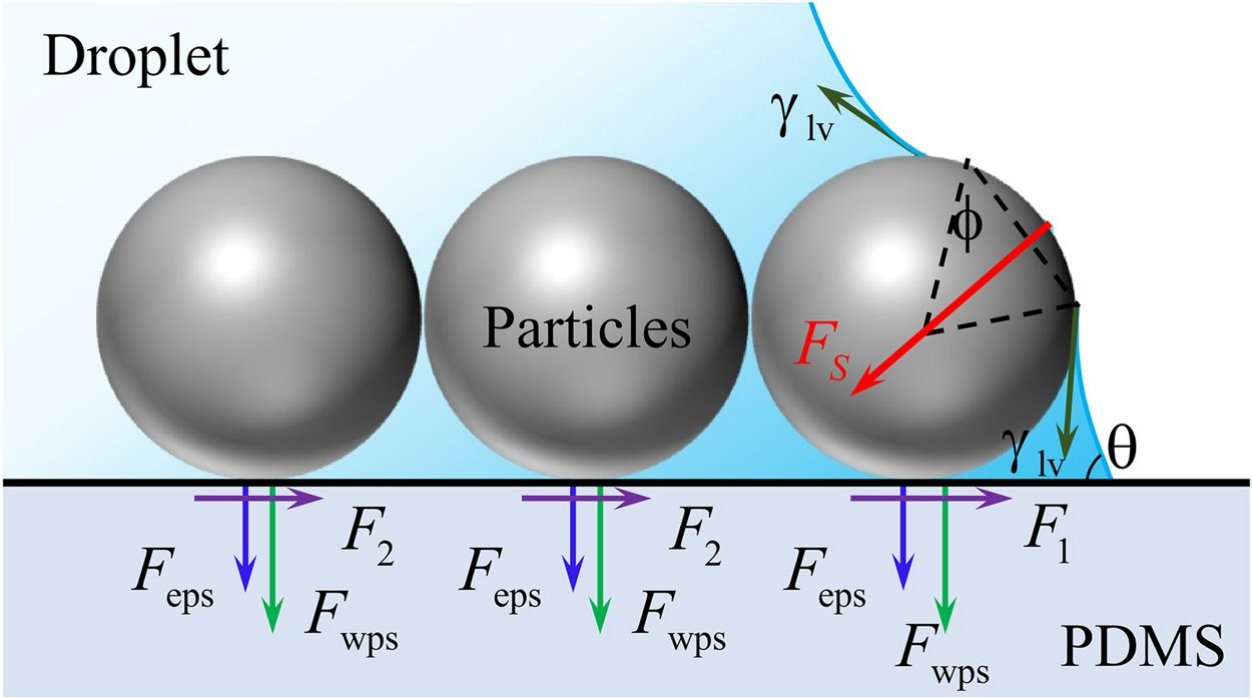
Schematics of self-pinning mechanism of microparticles at the contact line. ${F}_{1}$ and ${F}_{2}$ are the friction forces acting on each of the microparticles.

**Figure 9:**
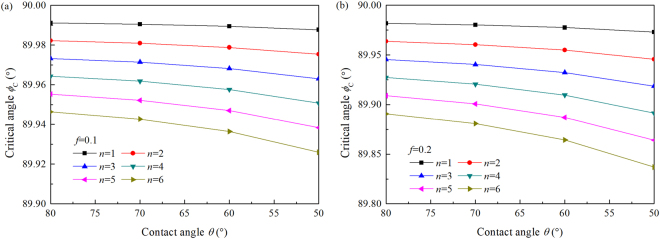
Critical angle depending on the number of microparticles: (**a)***f* = 0.1, (**b)***f* = 0.2.

In our previous paper^24^, we established a mechanism for the self-pinning of microparticles located at the contact line as (as shown in Fig. 8)

5 $${F}_{{\rm{S}}}\,\sin \,\theta -[f({F}_{{\rm{S}}}\,\cos \,\theta +n{F}_{{\rm{wps}}}+n{F}_{{\rm{eps}}})+n{F}_{{\rm{d}}}]=0$$

where *f* is the friction coefficient between the microparticle and the substrate in water or ethanol/water mixture (it should be noted that the coefficient is difficult to be determined up till now and it is assumed to be 0.1 or 0.2) and *n* is the number of microparticles located at the contact line in the radial direction. ${F}_{{\rm{wps}}}$ and ${F}_{{\rm{eps}}}$ are, respectively, the van der Waals and electrostatic forces between a microparticle and the substrate. ${F}_{{\rm{S}}}=2\pi R{\gamma }_{{\rm{lv}}}\,\cos \,\varphi$ is the capillary force acting on the outmost microparticle, where $\varphi$ is an angle to be determined and $(\pi -2\varphi )$ is the angle of the liquid layer covering the outmost microparticle, as shown in Fig. 9. Table 1 lists all the parameters for calculation of all the interaction forces in eq. (5) and Table 2 lists the values of these forces. Using eq. (5), the critical angle ${\varphi }_{{\rm{C}}}$ depending on the number $n$ was calculated, as shown in Fig. 9. It is found that there is only a thin liquid film acting on the outmost microparticle when it is self-pinned on the contact line.

**Table 1:** Parameters for calculation of interaction force acting on microparticles.

Symbol	Physical Parameter	Value	Unit
${A}_{{\rm{11}}}$	Hamaker constant of PS microparticle	$6.3\times {10}^{-20}$ ^44^	J
${A}_{{\rm{22}}}$	Hamaker constant of PDMS	$4.4\times {10}^{-20}$ ^44^	J
${A}_{{\rm{33}}}$	Hamaker constant of water	$3.7\times {10}^{-20}$ ^45^	J
${A}_{{\rm{132}}}$	Hamaker constant between the PS microparticle and PDMS in water	$0.10\times {10}^{-20}$	J
${\gamma }_{{\rm{lv}}}$	Surface tension of water	0.072	N/m
$z$	Minimum separation distance	$0.4\times {10}^{-9}$ ^20^	m
$\varepsilon$	Permittivity of water	$7\times {10}^{-10}$	F/m
${\varphi }_{1}$	Surface potential of PS	−59.5^46^	mV
${\varphi }_{2}$	Surface potential of PDMS	−45^47^	mV
$\eta$	Dynamic viscosity of water	0.0009	Pa·s
$v$	Velocity of water in evaporating droplet	1	μm/s
$\kappa$	Reciprocal of the Debye length	(430 × 10^−9^)^−1^ ^20^	m^−1^

**Table 2:** Values of some interaction forces.

Symbol	Equation	Value	Unit
${F}_{{\rm{wps}}}$	${F}_{{\rm{wps}}}=\frac{{A}_{132}R}{6{z}^{2}}$ ^19–21,48^	$1.06\times {10}^{4}$	pN
${F}_{{\rm{eps}}}$	${F}_{{\rm{eps}}}=-2R\varepsilon \kappa \frac{[{{\varphi }_{1}}^{2}+{{\varphi }_{2}}^{2}-2{\varphi }_{1}{\varphi }_{2}\exp (\kappa z)]}{[\exp (2\kappa z)-1]}$ ^19–21,49^	$-3.65\times {10}^{3}$	pN
${F}_{{\rm{d}}}$	${F}_{{\rm{d}}}=6\pi R\eta u$ ^42,42^	$0.17$	pN
${F}_{{\rm{S}}}$	${F}_{{\rm{S}}}=2\pi R{\gamma }_{{\rm{lv}}}\,\cos \,\varphi$ ^24^	To be determined	

For droplets with low microparticle concentration (the total number of microparticles inside the droplets is small), when the receding contact line approaches to the outmost microparticles, the liquid layer acting on the microparticles will become thicker and thicker, and there will be a very larger capillary force if the microparticles is still stationary. Meanwhile, the values of all the other forces do not increase. Thus Eq. (5) cannot hold and the microparticles have to move spontaneously together with the receding contact line under the action of capillary force, resulting in a compact monolayer structure. For a high microparticle concentration, on the one hand, the microparticles inside evaporating droplet is settling during the early stage, and there will be more microparticles settling near the center of the solid-liquid interface, meanwhile, similar to the case of low microparticle concentration, the outmost microparticles also move toward the center under the action of capillary force. Since there are more microparticles settling on the central zone of solid-liquid interface for this case, it is more likely to form a multi-layered compact structure. However, it should be noted that it is difficult to set up a self-pinning mechanism of multi-layered microparticles confined at the contact line.

## Methods

We prepared PDMS membranes for studying evaporation of sessile droplets containing PS microparticles. PDMS (Sylgard 184, Dow Corning, USA; mass ratios of base to curing agent = 10:1) was vacuumed for 30 minutes to remove the trapped air-bubbles and then spin-coated on the surface of clean glass surface at the rate of ~2000 rpm. Finally the samples were cured for about 8 hours at 80 °C. Particle suspensions were prepared by diluting PS suspensions (PS07N, mean diameter of particles: 20.33 ± 0.614 μm; Bangs Laboratories, Fisher, USA), from initial concentration of 9.9 wt. % to 0.02, 0.08, 0.32 and 1.28 wt. % in deionized water or ethanol/water mixture. The volume by volume concentration of ethanol in ethanol/water mixture was 0%, 10% and 20%.

0.60 ± 0.05 μL suspension droplet was deposited on the PDMS substrates using a micropipette. Before the deposition, the suspension was ultrasonically stirred for 10 min to ensure that the microparticles were homogeneously dispersed. Once the droplet was deposited onto the surface, OCA 20 system (precision: ± 0.1°, from Dataphysics, Germany) equipped with a high-resolution camera was immediately adjusted to record droplet evaporation at 0.5 fps. The environmental temperature and relative humidity (RH) are 23 ± 1 °C and 53 ± 3%, respectively. To ensure the reproducibility, each experiment was repeated six times. Finally, the deposition patterns after evaporation were measured using laser scanning confocal microscope (Keyence VK-X260, Japan).

## Conclusions

Evaporation of both water and ethanol/water mixture droplets containing larger PS microparticles was experimentally investigated. The evaporation of ethanol/water mixture droplet containing PS microparticles exhibits a different way from that of pure water droplet. When the concentration of microparticle is low, the contact radius decreases throughout the whole process while the contact angle increases at first to a maximum, then keeps almost constant, and finally decreases sharply. However, the evaporation of ethanol/water mixture droplet with higher concentration of microparticle behaviors more complex. The settling of microparticles inside the evaporating droplet was theoretically analyzed and compared with the experimental observation. At last, deposition patterns of microparticles were analyzed. It is found that at low microparticle concentration, a compact monolayer deposition of microparticles was obtained, while a multi-layer deposition pattern were formed for higher one. Small addition of ethanol brings a stronger flow inside evaporating droplet, yet has little influence on the deposition pattern.

## Electronic supplementary material

Supplementary movie 1

Supplementary movie 2

Supplementary movie 3

Supplementary movie 4
