# Author Correction: Evaporative deposition of polystyrene microparticles on PDMS surface

**DOI:** 10.1038/s41598-018-23132-9

**Published:** 2018-03-19

**Authors:** Ying-Song Yu, Ming-Chao Wang, Xianfu Huang

**Affiliations:** 1 Department of Mechanics, School of Civil Engineering, Architecture and Environment, Hubei University of Technology, Wuhan, 430068 China; 2 State Key Laboratory of Nonlinear Mechanics, Institute of Mechanics, Chinese Academy of Sciences, Beijing, 100190 China; 3 School of Engineering Science, University of Chinese Academy of Sciences, Beijing, 100049 China

Correction to: *Scientific Reports* 10.1038/s41598-017-14593-5, published online 26 October 2017

This Article contains errors in Equation 1.

$$V=\frac{\pi {a}^{3}{(1+\cos \theta )}^{2}(2\,-\,\cos \,\theta )}{3{\sin }^{3}\theta }$$

should read:

$$V=\frac{\pi {a}^{3}{(1-\cos \theta )}^{2}(2\,+\,\cos \,\theta )}{3{\sin }^{3}\theta }$$

